# Neoadjuvante Radiochemotherapie kombiniert mit regionaler Tiefenhyperthermie beim Rektumkarzinom?

**DOI:** 10.1007/s00066-021-01850-w

**Published:** 2021-09-14

**Authors:** Oliver J. Ott

**Affiliations:** Universitätsklinikum Erlangen, Universitätsstr. 27, 91054 Erlangen, Deutschland

## Hintergrund und Ziel

In der vorliegenden prospektiven Phase-II-Studie wurde der Einfluss einer additiven regionalen Tiefenhyperthermie im Rahmen einer neoadjuvanten Standardradiochemotherapie (RCT) beim lokal fortgeschrittenen Rektumkarzinom untersucht.

## Patienten und Methoden

Die Patient*innen erhielten eine 5‑Fluorouracil(5-FU)-basierte, konventionell fraktionierte präoperative RCT bis zu einer Gesamtdosis von 50,4 Gy. Zusätzlich wurde zweimal pro Woche eine regionale Tiefenhyperthermie durchgeführt. Postoperativ wurde der Effekt der neoadjuvanten Therapie mit dem Tumorregressionsscore nach Dworak graduiert. Der primäre Endpunkt war die pathologisch komplette Remission (pCR). Weitere Endpunkte waren die lokale sowie die distante Kontrolle, das krankheitsfreie (DFS) und das Gesamtüberleben (OS). Die additive Hyperthermie wurde als durchführbar bewertet, wenn 70 % der Patient*innen ≥ 8 Behandlungen während der Bestrahlungsserie erhalten hatten. Die Lebensqualität wurde mit den EORTC-QLQ-C30- und QLQ-CR29-Fragebogen untersucht. Die Studie war bei clinicaltrials.gov registriert (NCT02353858).

## Ergebnisse

Zwischen 2012 und 2017 wurden insgesamt 78 Patient*innen eingebracht. Die mediane Nachbeobachtungszeit betrug 54 Monate. Ein positiver oder naher (< 1 mm) CRM-Status lag in 60 % der Fälle vor. 99 % erhielten eine voll dosierte Radiotherapie, 91 % beide 5‑FU-Zyklen und 77 % erhielten ≥ 8 Hyperthermiebehandlungen. Die pCR-Rate betrug 14 % und ein Dworak-Tumorregressionsgrad 3–4 lag in 50 % der Fälle vor [1]. Nach drei Jahren waren die Raten für Gesamtüberleben, krankheitsfreies Überleben, lokale sowie distante Kontrolle 94 %, 81 %, 96 % bzw. 87 %. Höhere kumulative Temperaturen bei der Hyperthermie führten zu ausgeprägteren Tumorregressionen. Die Lebensqualitätsanalyse zeigte keine wesentlichen Abweichungen zur allgemeinen Bevölkerung.

## Schlussfolgerung der Autoren

Die regionale Tiefenhyperthermie war durchführbar, ohne die anderen Therapiemaßnahmen zu beeinträchtigen, und zeigte vielversprechende Verbesserungen hinsichtlich der onkologischen Endpunkte und der Lebensqualität.

## Kommentar

*Ja,* es handelt sich tatsächlich um eine prospektive Studie zur Effektivität der additiven Hyperthermie zur Radiotherapie bzw. RCT [2]. Und *ja,* es gibt auch heute noch klinisch tätige Wissenschaftler, die die Wirksamkeit einer additiven Hyperthermie im Rahmen multimodaler onkologischer Standardkonzepte untersuchen und die Hyperthermie nicht dem häufig unbegründeten und missbräuchlichen Einsatz im alternativmedizinischen Segment überlassen. In den letzten Jahren wurden mehrere retro- und prospektive Studien publiziert, bei denen die Hyperthermie im direkten randomisierten oder historischen Vergleich zu einer Verbesserung relevanter klinischer Endpunkte führte [3–6]. Trotzdem wird die Hyperthermie als Radiosensitizer weithin unterschätzt.

In der vorliegenden prospektiven Phase II-Studie wurde eine 5‑FU-basierte neoadjuvante Standard-RCT mit bis zu 10 regionalen Tiefenhyperthermiebehandlungen ergänzt. Die Compliance der Patient*innen war hoch. Und bezüglich Toxizität, Lebensqualität und der üblichen onkologischen Endpunkte fanden sich erwartungsgemäß ähnliche Ergebnisse wie in Studien ohne additive Hyperthermie. Die Rate an histologisch gesicherten kompletten Remissionen (pCR) betrug 14 % und der Tumorregressionsgrad 3–4 nach Dworak wurde in 50 % der Fälle erreicht. Interessant ist, dass höhere kumulative Hyperthermietemperaturen mit einer signifikant besseren Tumorregression korrelierten.

Hinweise, dass eine additive Hyperthermie in Verbindung mit einer Radiotherapie beim lokal fortgeschrittenen Rektumkarzinom zu einer besseren Tumorrückbildung führen kann, wurden bereits 2009 in einer Cochrane-Database-of-Systematic-Reviews-Metaanalyse beschrieben [7]. Bei der Auswertung von 6 randomisierten Studien mit insgesamt 520 Patient*innen fand sich mit Hyperthermie auch ein Vorteil im Gesamtüberleben nach 2 Jahren, der allerdings nach 5 Jahren nicht mehr nachweisbar war. Zusätzlich kam es mit Hyperthermie zu einer hochsignifikant höheren Rate an kompletten Tumorremissionen im Vergleich zur alleinigen Bestrahlung. Das war der erste klare Hinweis, dass eine zusätzliche regionale Tiefenhyperthermie im Rahmen eines multimodalen neoadjuvanten Konzepts weitere therapeutische Vorteile bringt und deshalb gut begründet ist. Auf der Basis dieser Daten wurde auch die prospektive und multizentrische HyRec-Studie initiiert und durchgeführt [4]. Auch bei dieser Phase-II-Studie konnte die neoadjuvante 5‑FU- und Oxaliplatin-basierte Radiochemotherapie mit einer sehr hohen Protokolladhärenz bei 105 Patient*innen mit einem lokal fortgeschrittenen oder lokal rezidivierten Rektumkarzinom durchgeführt werden. Hier waren ebenso wie in der Tübinger Studie 10 konkomitante regionale Tiefenhyperthermiebehandlungen vorgesehen. Obwohl sich das Studienkollektiv im Vergleich zur Tübinger Studie noch etwas ungünstiger zusammensetzte (16/105 Rezidivpatient*innen, zusätzlich Patienten mit Oligometastasierung, UICC-Stadium II ausgeschlossen etc.), betrug die pCR-Rate 19 % und die Tumorregressionsgraduierung nach Dworak 3–4 68 % in Bezug auf die vorliegenden Befunde.

Was macht man jetzt mit diesen Ergebnissen? Welchen Beitrag könnte die additive regionale Tiefenhyperthermie zukünftig in der Versorgung von Patient*innen mit einem Rektumkarzinom einnehmen? Erstens bleibt festzuhalten, dass eine additive regionale Tiefenhyperthermie mit bis zu 10 Fraktionen während einer 5‑FU- oder 5‑FU/Oxaliplatin-basierten Radiochemotherapie sicher durchführbar ist. Zweitens darf man einen therapeutischen Nutzen durch die additive regionale Tiefenhyperthermie immer dann annehmen, wenn die größtmögliche Tumorrückbildung quoad vitam entscheidend ist. So können heute 25–50 % aller Patient*innen mit einem Lokalrezidiv erneut kurativ behandelt und tatsächlich auch geheilt werden [8]. Zwingende Voraussetzung dafür ist jedoch das Erreichen einer kompletten Resektion (R0). Diese Erkenntnisse sind deshalb nicht hoch genug einzuschätzen, weil es zur Behandlung von Rezidiven bisher kaum prospektiv erhobene Daten gibt, die mit dem multimodalen Therapieansatz der HyRec-Studie verglichen werden dürfen. Somit wurde ein Therapiekonzept für Lokalrezidive gut untersucht und etabliert, welches die regionale Tiefenhyperthermie vollgültig einbezieht. Es dürfte wohl auch jedem klar sein, dass es wegen des seltenen Vorkommens lokal rezidivierter Rektumkarzinome in absehbarer Zeit wohl keine randomisiert erhobenen Daten geben wird, die die hier vorgestellten Ergebnisse ergänzen können.

## Fazit

Die regionale Tiefenhyperthermie hat sich in den letzten Jahren auf dem wissenschaftlichen Parkett gut präsentiert. Sie wird, eine sinnvolle und strahlenbiologisch begründete Patient*innenselektion vorausgesetzt, auch in Zukunft das Vertrauen der Kostenträger rechtfertigen und weitere positive Ergebnisse zur onkologischen Versorgung beitragen.

Ein weiteres zukünftiges Einsatzgebiet der additiven regionalen Tiefenhyperthermie könnte sein, durch eine weitere Steigerung der pCR-Rate die heute noch obligate Operation in einem größeren Prozentsatz der Betroffenen mit einem lokal fortgeschrittenen Rektumkarzinom zu vermeiden. In dieser Beziehung besteht allerdings noch ein enormer Forschungsbedarf, und zwar nicht nur hinsichtlich der Hyperthermie.

*Oliver J. Ott, Erlangen*
